# Medical student perceptions of gender and pain: a systematic review of the literature

**DOI:** 10.1186/s12916-024-03660-0

**Published:** 2024-10-08

**Authors:** Maia Patrick-Smith, Stephanie Bull

**Affiliations:** Imperial College London, Exhibition Rd, South Kensington, London, SW7 2AZ UK

**Keywords:** Gender, Pain, Medical students, Medical education

## Abstract

**Background:**

Gender bias exists in healthcare and affects how pain is assessed and managed. This bias affects patient outcomes and their trust in healthcare professionals. We also know that future clinicians develop their attitudes early in training. Medical school is therefore an opportunity to shape the values of future doctors and to combat systemic gender bias in healthcare. This systematic review aims to explore medical student perceptions of the relationship between patients’ gender and their pain, so that recommendations can be made for developing medical education.

**Methods:**

Embase, MEDLINE, PsychINFO, LILACS, Global Index Medicus, PakMediNet and ERIC were searched for articles relating to medical student perceptions of gender and pain, with no geographical or language limitations. Quality was assessed using the Medical Education Research Study Quality Index and the Critical Appraisal Skills Programme checklist.

**Results:**

Nine publications were identified, two qualitative and seven quantitative. All studies had methodological limitations. Many different study designs were used, although most involved simulated patients. All studies referred to gender as binary. Multiple studies found that women’s pain is more likely to be underestimated by medical students and that the patient’s gender drives different approaches during clinical history taking, examination and management in these simulated situations. Only one study found no effect of patients’ gender on students’ perception of their pain.

**Conclusions:**

Whilst there is a paucity of high-quality studies in this area, patients’ gender was found to affect how their pain is perceived by medical students. No studies explored where students’ attitudes towards gender and pain arise from, and few involved ‘real life situations’. We propose that further work into medical student perceptions in ‘real situations’ is needed. This will help to inform how undergraduate medical education can be developed to tackle gender bias, and ultimately improve outcomes for patients.

**Supplementary Information:**

The online version contains supplementary material available at 10.1186/s12916-024-03660-0.

## Background

It is well established that there is a gender bias in the assessment and management of pain by healthcare professionals [1–4]. Women’s pain is systematically underestimated by clinicians and undertreated in comparison to pain experienced by men [1]. Women have been found to receive less analgesia after surgery [5] and are more likely to be prescribed psychological treatment rather than traditional analgesia for their pain [3]. This is on a background of significant pain-related morbidity in women, with one in five women in the USA living with chronic pain [6]. The difference in the way that pain is assessed and managed based on a patient’s gender results in women receiving inadequate pain management [5, 7] and it taking women longer to receive a diagnosis than men for the same types of pain [8]. This impacts on their immediate and long-term health, as well as their trust in healthcare professionals [4].

Two main types of gender bias are seen in medicine: *gender stereotyping*, where patients are treated differently because of their gender in a clinically unjustified way; and *gender blindness*, where clinically important differences between the genders are overlooked [9, 10]. Though we know that both forms of bias play a role in how doctors respond to and manage pain, what is less clear are the origins of these biases [11].

Medical students are the doctors of the future and medical school, as well as medical school faculty and the wider clinical team that students encounter, shapes many of their professional values and perspectives [12]. This may be through formal learning from the official medical school curriculum, or through informal learning which can be termed the ‘hidden curriculum’ [13]. There have been multiple calls for greater discussion of gender bias in medical school curricula [9, 10], and some medical schools have already developed tools to help students improve their gender awareness [14, 15]. However, without a greater understanding of the pre-existing attitudes and perceptions amongst medical students, and of whether they hold similar attitudes to clinicians in relation to patients’ gender and their pain, it is difficult to determine where efforts should be targeted, and which interventions will have the best chance of success.

The aim of this study is to conduct a systematic review of the literature [16] to explore medical student perceptions of the relationship between patients’ gender and their pain. We are seeking to understand what is known, what remains unknown and where there is uncertainty around findings. The ambition is that by understanding the current state of knowledge, we can make recommendations for what should come next, so that we can develop medical education that better supports the reduction of gender bias in medical students, our future doctors, and ultimately improve patient care.

## Methods

The systematic review approach followed PRISMA guidelines ( see Additional file 1: Supplementary file 1 and Additional file 2: Supplementary file 2) and is described below.

### Search

MP-S and SB created the initial search to identify articles relating to medical students’ perception of the relationship between a patient’s gender and their pain, considering the wide range of keywords and Medical Subject Headings (MeSH) that could be used to describe the concepts of gender, pain and medical students. Search testing was performed by MP-S in Embase. This involved reviewing the dominant MeSH terms used to tag 3 known relevant articles and adapting the search so that these were included. Further refinement to the MeSH terms and keywords were made to reflect this.

Keywords, and their Boolean combinations, were used across databases Embase, MEDLINE, PsychINFO, LILACS, Global Index Medicus, PakMediNet and ERIC. MeSH terms were available in MEDLINE, Embase, PsychINFO, LILACS, Global Index Medicus and ERIC and were varied to reflect the vocabulary of each database. PakMediNet does not use MeSH terms, and so keywords were used only.

Searches were conducted on 18 May 2023. The search string for Embase is displayed below with MESH terms indicated in bold:

(**Medical School** OR **Medical Student** OR **Medical Education** OR BMBS OR MBBS OR BMBCh OR medic* adj2 student*) AND (**Gender** OR **Gender Equity** OR **Gender Inequality** OR gender* OR **Gender Bias** OR **Sexism**) AND (**Pain** OR **Pain Assessment** OR **Pain Intensity** OR **Pain Severity** OR **Chronic Pain** OR **Pain Threshold** OR **Psychogenic Pain** OR pain* OR **Analgesia**).

The search string was translated into the syntax and vocabulary of each additional database (Additional file 3: Supplementary file 3).

The results from each database search were imported into Endnote 21 (Clarivate PLC, Jersey, UK) for ease of de-duplication. After de-duplication, studies were uploaded to a screening platform Covidence (Veritas Health Innovation, Melbourne, Australia) to facilitate team collaboration whilst screening. Titles and abstracts of studies were screened against the listed inclusion and exclusion criteria by one reviewer (MP-S). At this stage, a cautious approach was adopted, only excluding articles that obviously bore no relation to the research question.

### Study selection

The following inclusion and exclusion criteria were used:

- Studies involving medical students but not qualified healthcare professionals or students studying any other healthcare discipline.
- Studies where it was not possible to separate outcomes or analysis of medical students’ perceptions from other groups of healthcare professionals were excluded.
- Studies looking at medical students’ perception of (attitudes towards, opinions on, experience of, or evaluations of) the relationship between a patient’s gender and their pain (assessment and/or management).
- Studies that did not focus on the medical students’ *perception* of gender and pain (e.g. studies solely looking at the prevalence of pain between genders) were excluded.
- Studies published in any language—translation to the English language was conducted using Google Translate.
- Studies from any geographical location.
- Qualitative or quantitative empirical studies, opinion or comment pieces and conference proceedings were included.
- Literature indexed within databases: Embase, PsychINFO, ERIC, MEDLINE, Global Index Medicus, PakMediNet and LILACS.
- The reference lists of all included published studies were also searched.

**Fig. 1:**
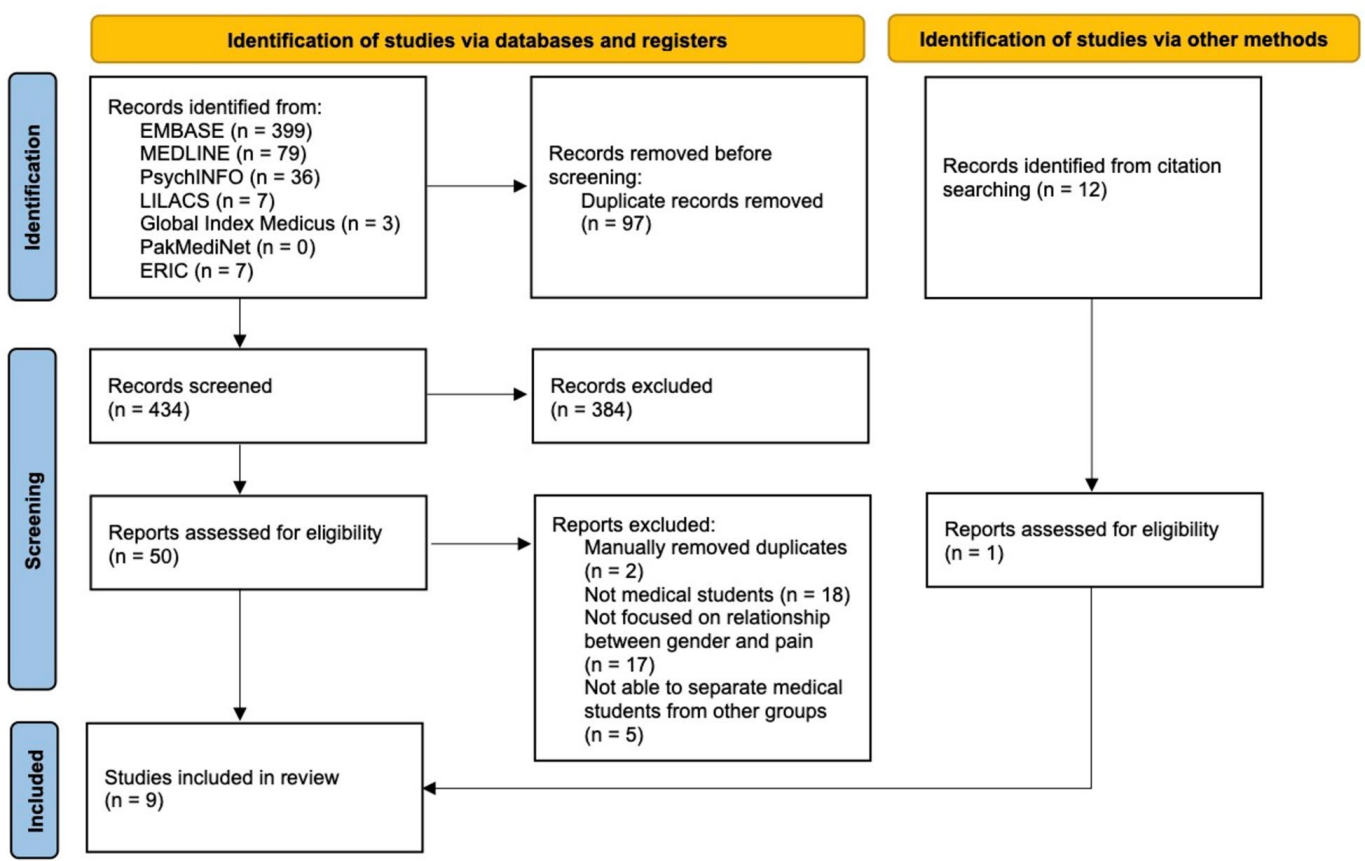
Flow diagram (based on PRISMA template [19]) showing the data collection process for the included studies

Three people (MP-S, SB and MB — see the ‘Acknowledgements’ section) reviewed the full text of the articles against the inclusion criteria. MP-S and SB met to discuss any uncertainties over inclusion, adding clarifying details to the inclusion and exclusion criteria to enhance transparency. Uncertainties over inclusion were discussed by two reviewers (MP-S and SB) until a consensus was formed. The reference lists of included articles were screened by MP-S to search for any relevant articles that had not been identified in the preliminary search. One study required translation from French which was performed using Google Translate (Fig. 1).

### Data extraction

Data was extracted from each included paper by MP-S and SB. Data extracted included paper identifiers (authorship, date), study type (empirical qualitative, quantitative), study setting (geographical location and type of setting), the aims of the study that were relevant to the research question and details of the study including methods, results and conclusions (in relation to medical students perceptions of gender and pain) that can be drawn from each study.

### Quality assessment

The quality of included empirical studies involving quantitative and qualitative research was assessed by one reviewer (MP-S), using the Medical Education Research Study Quality Index (MERSQI) [17] and the Critical Appraisal Skills Programme (CASP) checklist [18]. This checklist is not designed to assign a numerical score to assess quality and has been used to identify where there are perceived methodological limitations. Uncertainties were resolved through discussion with SB.

### Synthesis

A descriptive synthesis of studies was conducted taking care to address the main research question: how do medical students perceive the relationship between patients’ gender and their pain?

### Reflexivity

Whilst the authorship team have conducted a systematic search and synthesis of the evidence, they would also like to share their positionality with regard to the research topic. Both identify as women, are of white ethnicity and have roles as an educator and an early career clinician who both teach medical students. Both SB and MP-S have experienced pain requiring assessment and treatment by healthcare professionals. Each was satisfied with the pain management that they received and felt that it was not affected by their gender. The researchers took time to reflect on whether their experiences may be influencing their interpretation of the findings during the research process.

## Results

Nine empirical studies met the inclusion criteria (Additional file 4: Table 1), made up of two qualitative studies [15, 20] and seven quantitative studies [2, 21–26]. No review articles, opinion or comment pieces were found during the search. The quantitative studies ranged in size, with two studies having less than 30 participants [2, 25], one with over 200 participants [22] and the remainder between the two. Methodological limitations were identified in all publications during the quality appraisal process, although there was a variation in quality across the studies (MERSQI score: 9.5–12.5, CASP: 1–5 limitations). A cut-off MERSQI score of 14 out of a possible 18 has been used to indicate high quality in previous literature [27]. Notably, in many of the studies, the role of gender in relation to pain was not the main focus of the research, rather it was a variable that was being taken into consideration in a study with other goals. All studies were from the Global North [28], with three from the USA, two from the UK and four from mainland Europe. Seven studies were based at a single site and two were multi-site studies [21, 22].

### Study setting

The two qualitative studies [15, 20] looked at student reflections of real clinical cases that they had observed, whereas all other studies used simulations—either actors, mannikins or written vignettes—where the student acted as the clinician. Two studies [21, 22] considered the perception of gender and pain in a paediatric patient population and the rest considered the relationship between gender and pain in adults. Six studies looked at acute pain [15, 22–26], two used examples of chronic pain [2, 21] and one had a mixed sample [20]. All studies referred to gender as binary and none clarified whether they referred to gender identity or biological sex when using terms such as ‘man/men’, ‘woman/women’, ‘boy’, ‘girl’, ‘male’ or ‘female’ within their manuscripts.

### Perceptions and biases

Eight of the nine studies concluded that gender influenced student’s perceptions of patient’s pain, measured through assigned pain ratings or through student reflections. In the only study that did not make this association, the patient population were children undergoing venepuncture [22]. Notably, the study used a video of a child that had been ‘validated’ to have a gender-neutral appearance, and the authors assigned the child a typically male or typically female name within each vignette to denote the child’s gender. Two studies found that medical students assigned women lower pain ratings than men despite the identical nature of the other aspects of the case [2, 26]. Another showed that medical students felt that, in children, girls were more distressed by their pain than boys. The same study also tested medical students’ attitudes towards gender and pain using an implicit association test (designed for measuring attitudes towards adults in pain) and found that students perceive women to be more pain-sensitive than men [21]. One qualitative study described a student reflection of a clinical encounter where the attitude held by medical professionals was that men express pain less than women [15]. The student felt this had affected the quality of care that the man was given as non-pharmaceutical options to alleviate pain were not explored.

### Clinical history, examination and investigations

A gender bias in the clinical history-taking and examination of patients with pain was found in three studies. One study found that the characteristics of a patient’s pain were explored more thoroughly by medical students when the patient was a woman [23] and in another qualitative study, a student reflected that women in pain may be examined differently by clinicians due to concerns about modesty or dignity [20]. More subtle gender differences were revealed in a study that showed that the students’ own gender influences the ability to interpret pain expressions in patients of the opposite gender when there is a disconnect between the student’s belief in the pain level and the patient’s (in this case a mannikin) response to pain. This altered their examination technique with students palpating the abdomen more forcefully when the patients’ pain was disbelieved [25].

The only study that looked at investigations for patients with pain found no difference in the frequency with which computed tomography (CT) scans were proposed for patients who were men and women with symptoms of acute aortic dissection during an Observed Structured Clinical Examination [23].

### Diagnosis and management

The relationship between pain and psychological distress was explored in two publications [23, 24]. One study found that, in the context of stress and anxiety, women’s pain was more likely to be perceived as psychogenic than men’s pain [24]. Another study identified that for patients with identical presentations, anxiety was more frequently diagnosed as the causative complaint in women than men [23].

Students reflected that in circumstances where women’s pain is believed to be psychological, then less analgesia is likely to be given and there can be a potential delay in diagnosis [20]. Another student reflected that women may be more likely to be offered non-pharmacological interventions for their pain than men [15]. In a quantitative study, however, despite perceiving a gender difference in the level of distress that a child displayed whilst in pain, there was no difference in how the medical student treated the patient [21].

## Discussion

We know that healthcare professionals’ perception of patients’ pain is influenced by patient gender; however, it was not previously clear whether medical students hold similar perceptions with regard to patients’ gender and their pain, and if so, how, why and when these perceptions develop.

This review has shown that medical students do hold the perception that women are more pain sensitive than men and that they regard women to feel more distressed and to be inclined to exaggerate the level of pain that they feel. However, we also identified that there is a paucity of literature specifically on medical student perceptions of the relationship between patients’ gender and their pain and that the studies that have been conducted are of relatively low methodological quality. Therefore, a cautious approach should be adopted when interpreting study findings, and more studies addressing this research area would be beneficial. All but two studies occurred in simulated situations, meaning that it is unclear how these perceptions may play out in real-life clinical encounters. In addition, whilst it is thought that gender can affect all aspects of a patient’s pain-related care, from the assessment (history-taking, examination and investigations) to management, there was only one study that had looked at the impact of gender on medical students approach to ordering clinical investigations [23]. This is another gap in the literature where further work would be useful.

There are many examples of how gender influences patient care. Women are underrepresented in research trials, which results in gaps in our knowledge about women’s health and means that it is often assumed that conditions present in the same manner in both men and women. Men are more likely to experience left-sided chest pain when having a ‘heart attack’ or myocardial infarction (MI), and therefore the symptoms that women are more likely to experience such as nausea or abdominal discomfort are labelled as ‘atypical’. This is likely to explain why, in the case of MI, there is often a delay in women presenting to the hospital and receiving treatment [29]. When we consider some of the outcomes in clinical practice that may be specifically associated with gender bias and pain, we see that women are more likely to be prescribed antidepressants or receive a mental health referral for pain symptoms than men [3, 4, 11] and that women presenting with chest pain are less likely to receive diagnostic and interventional cardiac procedures [30]. We also know that women report feeling disbelieved by physicians [31] and that this disbelief can lead to a breakdown of the therapeutic relationship and can cause worsening of the patient’s pain [32]. It is therefore of particular interest that we also found that when features of stress and anxiety were added to vignettes of patients with chest pain, medical students deemed women more likely to have a psychogenic cause of their pain whereas no effect was seen with men [24]. It was also noted that medical students asked women more in-depth questions about their pain than men [23] and whilst the reason for this was not explored further, might it represent a lack of belief in the woman’s pain and a feeling that women’s pain is more likely to be driven by poor mental health rather than having a physical basis? It has been seen that medical students assess patients’ pain levels as higher when there is clear medical evidence to support why a patient is in pain [33].

We know that perceptions of gender are complex and that gender roles within our society exist. Such roles may mean that a stereotypical man is seen as stoic and less emotional [34], and a stereotypical woman is seen as dramatic and irrational [35] and as having a greater tendency to overtly express their pain [36]. This can then affect the way that their pain is perceived by others [1, 37]. It may also be that the gender bias experienced by a patient in relation to their pain is affected by the extent to which they display more typically ‘masculine’ or ‘feminine’ traits within their gender identity. Factors other than gender are known to affect the perception of pain by clinicians, the most studied demographic in this context being ethnicity [38]; however, this review has focused solely on gender. In children, the influence of gender in relation to pain perception by clinicians appears to be less strong and strengthens during adolescence [39]. It is interesting that the only study in this review where students did not express gender bias [22] involved a child patient with a gender-neutral appearance. As the aim of this review was to document what is known about the perception of pain by medical students according to patient gender and where the gaps are, it was important to include patients from any demographic.

Whilst existing studies show that gender does affect the way pain is perceived by medical students, there is limited explanation of why this is the case and how, why and when these views have developed. As educators, we are particularly interested to understand the influence of medical training on these perceptions. Further research is required in this field, and in particular explorations of how students’ values and perspectives are shaped by both the formal and informal medical curriculum. Further exploration of this topic is important as medical students are our future clinicians and we know that medical school has an impact on the values that they hold [12]. Medical school is a timely opportunity to shape these values and although we now know that medical students do perceive pain differently depending on the patient’s gender, at present there is not enough research looking into where these attitudes come from. With this piece of the puzzle missing, it becomes difficult to know how best to integrate learning on this topic into teaching.

### Strengths

This is the first published literature review on this subject to our knowledge. Multiple databases were searched, and care was taken to broaden the search beyond the Global North by including databases indexing literature with a wider geographical reach. The search strategy and inclusion criteria were developed in an iterative fashion, with continuous testing, and regular discussion between the authors. The authors also spent time reflecting on how their own experiences and perceptions may affect their interpretation of the research findings.

### Limitations

Despite the care taken to conduct an exhaustive search, the included studies remained geographically limited and as such their findings are bounded in the values and cultures of those conducting the research. The methodological quality of the studies identified was generally low, and so our interpretations of the findings need to be viewed within this context. This review has not taken an intersectional approach and does not consider other variables that may affect medical students’ perceptions of patients’ pain beyond gender, such as ethnicity and social class. At present, there is not enough literature in the field to be able to take an intersectional approach; however, this would be an important future work. The study also did not use explicit search terms to include patients with transgender or non-binary gender identities, although the MeSH term ‘Gender’ was included across all databases. Research into how biological sex affects pain has been researched by many teams over the last 40 years [40, 41]. It is therefore possible that some of the medical students within the studies we have included have been exposed to this research and that their perception of gender and pain has been affected by this exposure. This was not explored within this review but would be an interesting topic for future research. Despite the careful reflection carried out by the authorship team throughout the research process, the interpretations may be limited by the experiences, ethnicity and gender of the authors.

## Conclusions

Existing studies have shown that medical students’ perception of pain is influenced by the gender of the patient. The biases seen in medical students align with those that are seen in clinical practice and are seen across all aspects of the patient journey, including both the assessment and management of a patient’s pain. The evidence base is, however, weak and gaps remain in our knowledge, particularly with relation to how, why and when medical students develop these biases. Further work addressing these questions and ultimately considering pain perception in a more intersectional context would be valuable. Understanding more about medical students’ perception of pain and gender is key to the planning and implementation of effective learning within the undergraduate medical school curriculum.

## Supplementary Information

Additional file 1.

Additional file 2.

Additional file 3.

Additional file 4.

## Data Availability

Data sharing is not applicable to this article as no datasets were generated or analysed during the current study.

## Abbreviations

CASP: Critical Appraisal Skills Programme
CT: Computed tomography
MERSQI: Medical Education Research Study Quality Index
MeSH: Medical Subject Heading
MI: Myocardial infarction

## Data availability

No datasets were generated or analysed during the current study.
