# Supplementary material for: Medical student perceptions of gender and pain: a systematic review of the literature
Source: BMC Med. 2024 Oct 8;22:434. doi: 10.1186/s12916-024-03660-0 (PMC11463120; doi:10.1186/s12916-024-03660-0)
Supplement: Supplementary file 3 — Additional file 3. [file 12916_2024_3660_MOESM3_ESM.docx]

Additional File 1: Supplement 1: Search Strings

Searches were conducted on 18th May 2023. The search strings for each database are displayed below with MESH terms indicated in bold:

| Category (each joined with AND) | MEDLINE | EMBASE | PsychINFO | LILACS & Global Index Medicus | PakMediNet | ERIC |
| --- | --- | --- | --- | --- | --- | --- |
| Medical Student | **Schools, Medical** OR **Students, Medical** OR **Education, Medical** OR BMBS OR MBBS OR BMBCh OR medic* adj2 student* | **Medical School** OR **Medical Student** OR **Medical Education** OR BMBS OR MBBS OR BMBCh OR medic* adj2 student*) | **Medical Students** OR **Medical Education** OR BMBS OR MBBS OR BMBCh OR medic* adj2 student* | "**Students, Medical**" OR "**Schools, Medical**" OR "**Education, Medical**" OR (medic* adj2 student*) OR BMBS OR MBBS OR BMBCh | medic* adj2 student* OR BMBS OR MBBS OR BMBCh | **medical students** OR **medical education** OR **medical school OR** (medic* adj2 student*) OR BMBS OR MBBS OR BMBCh |
| Gender | **Gender Equity** OR **Gender Role** OR gender* OR **Sexism** | **Gender** OR **Gender Equity** OR **Gender Inequality** OR gender* OR **Gender Bias** OR **Sexism** | **Gender Gap** OR **Gender Equality** OR **Gender Roles** OR **Gender Role** **Attitudes** OR Gender* OR **Sexism** | **"Gender Role"** OR **"Sexism"** OR **"Gender Equity"** OR gender* | gender* | **gender** OR **gender roles** OR **gender differences** OR **gender inequality** OR **gender equality** OR **gender bias** OR gender* OR **sexism** |
| Pain | **Pain** OR **Pain Measurement** OR **Pain Perception** OR **Acute Pain** OR **Chronic Pain** OR **Pain Threshold** OR **Pain Management** or pain* | **Pain** OR **Pain Assessment** OR **Pain Intensity** OR **Pain Severity** OR **Chronic Pain** OR **Pain Threshold** OR **Psychogenic Pain** OR pain* OR **Analgesia** | **Pain** OR **Pain Measurement** OR **Acute Pain** OR **Pain Perception** OR **Chronic Pain** OR **Pain Thresholds** OR **Pain Management** OR **Analgesia** OR pain* | "**Pain**" OR "**Pain Measurement**" OR "**Pain Threshold**" OR "**Pain Management**" OR "**Acute Pain**" OR "**Chronic Pain**" OR "**Analgesia**" OR pain* | pain* | **pain management** OR **managing pain** OR **analgesia OR** **pain assessment** OR **pain threshold** or **pain tolerance** OR **pain severity** or **pain intensity** OR **acute pain** OR **chronic pain** OR **pain management** OR pain* |
