# Supplementary material for: Medical student perceptions of gender and pain: a systematic review of the literature
Source: BMC Med. 2024 Oct 8;22:434. doi: 10.1186/s12916-024-03660-0 (PMC11463120; doi:10.1186/s12916-024-03660-0)
Supplement: Supplementary file 4 — Additional file 4. [file 12916_2024_3660_MOESM4_ESM.docx]

| **Paper identifier** | **Study type**  A= empirical qualitative  B = empirical quantitative | **Study Setting**  (geographical location, type of setting) | **Study’s Relevant Aims** | **Details**  (how this relates to our RQ, research methods) | **Results** | **Conclusions** | **No of limitations** **identified through CASP checklist if** qualitative study.  **Quality of studies assessed using MERSQI if** quantitative study |
| --- | --- | --- | --- | --- | --- | --- | --- |
| Geiser et al, 2022 | A | Switzerland, single-site, observation of real clinical encounters | To understand whether integrating reflection in clinical practice increases awareness of gender bias in medical school teaching | 160 medical students, working in small groups, presented a clinical case that they had observed in practice. Each group reflected on the influence of gender in each case, facilitated by an academic with expertise in gender medicine. Each student reflected individually on their gender biases, and on what they learned about gender bias and gender medicine within the session, recording their responses in a questionnaire. | Students felt that often women were given insufficient analgesic doses or received delays in diagnosis.  One student reflected that they believe a patient was not prescribed stronger analgesia as her pain was deemed to be ‘psychological’. The student felt that this was due to gender bias and that it had contributed to a significant delay in diagnosis.  Another student reflected that a patient with lumbar pain was not examined sufficiently as the doctor did not ask the patient to remove her clothes. The student felt that the patient may have been examined differently if they had been a man. | Students reflected that patients with pain may be examined and managed differently by clinicians according to gender, with women often being given less analgesia and being diagnosed later than men. | 1 |
| Geiser et al, 2020 | A | Switzerland, single-site, observation of real clinical encounters | To promote and record students’ reflections on the influence of gender on pain in clinical practice | 4 medical students presented and reflected on clinical cases that they had observed, considering if the consultation would have been different if the patient had been a different gender.  Individual reflections and discussions between students were analysed. | One student discussed a man receiving sutures to close a wound who looked faint but did not report this to the clinician. The same patient commented that he did not want any analgesia as a masculine man would receive treatment without analgesia.  The student reflected that women are more open to using relaxation strategies to alleviate discomfort and that clinicians may underestimate ‘vasovagal discomfort’ in men with very masculine appearances. They felt that the treatment of women should occur quicker and that pain in men should not be minimised. | Students reflected on stereotypical views that men minimise their pain, and that pain in woman may be treated by non-pharmaceutical approaches | 5 |
| Miller et al, 2020 | B | United States, multi-site, computer-simulated patients | To examine the effects of patient race and gender on medical students’ pain assessment and treatment decisions for children with chronic abdominal pain | 129 medical students viewed videos of computer-simulated paediatric patients (SPs) with chronic abdominal pain and were asked to make pain assessment and treatment decisions. SPs varied by race & gender but exhibited similar pain behaviours. Participants indicated the amount of distress, interference and overreaction they perceived the patients to be experiencing on a rating scale. Participants also indicated their likelihood of using a variety of treatment options for each patient. Participants completed 2 tests which assessed pain-related implicit attitudes about race and gender differences in pain. | Students rated girl SPs as more distressed by their pain than boys.  Students’ rating of SPs’ reaction to pain did not significantly differ by SP gender.  Decisions made by students about management (e.g., recommendations for opioid versus non-opioid medication, referral to specialist care, or provision of educational accommodations) did not differ according to SP gender.  Students implicit attitude tests to gender and pain demonstrated that men are perceived as being more pain-tolerant and women are more pain sensitive to a moderate extent.  An individual student’s implicit pain-related attitude about gender and pain did not predict their response to pain rating, management or treatment. | Medical students rated girls as more distressed by their pain than boys, however this did not affect their management decisions.  Students have moderate implicit attitudes that men are more tolerant, and women are more sensitive to pain. | 12.5 |
| Reardon et al, 2021 | B | United States, multi-site, simulated patients | To consider the influence of a child’s perceived gender on medical students’ assessment of the child’s level of pain. | 499 medical students viewed a video of a 5-year-old gender-neutral child undergoing venepuncture. They were told the child’s gender by the researchers. Students were asked to record on a pain rating scale how much pain the child experienced during the procedure. | Students’ pain ratings for the child undergoing the procedure did not significantly differ according to whether they were told that the child was a girl or a boy. | Students pain ratings of the child undergoing the procedure did not significantly differ according to gender. | 12.5 |
| Le Boudec et al, 2023 | B | Switzerland, single-site, simulated patients | To evaluate whether male and female patients are evaluated and treated differently when presenting with symptoms of Generalised Anxiety Disorder (GAD) or Acute Aortic Dissection (AAD) | 110 medical students took part in Observed Structured Clinical Examination (OSCE) stations that were constructed so that half of the participants encountered a female simulated patient (SP) experiencing GAD and AAD, and half a male SP (with all other characteristics preserved). Diagnosis and treatment decisions made by students were compared for male versus female patients. | Students are more likely to correctly diagnose GAD in women SPs than men but were equally likely to make the correct diagnosis with both genders with AAD.  Students were more likely to correctly identify the emergency nature of AAD in women SPs compared with men.  The frequency with which a CT scan was proposed or analgesia was given was not affected by the patient gender.  Pain characteristics were better explored when the SP with AAD was a woman. | Students’ assessment and management of SPs with GAD and AAD differed according to gender. | 12 |
| Chiaramonte & Friend, 2006 | B | United States, single-site, written vignettes | To explore the influence of social stress in a patient’s history on students interpretation of cardiac symptoms according to patient’s gender. | 82 medical students were given a written vignette about either a woman or man experiencing cardiac symptoms including chest pain. In half of the vignettes recent social stress was included and the patient appeared ‘nervous and agitated’. Participants received a questionnaire asking them to list the symptoms that were most important to their assessment of the patient and to indicate whether the aetiology of these symptoms were mostly or somewhat physical/organic or mostly or somewhat psychogenic. | When looking at patients without evidence of stress or anxiety, students interpreted the patient’s chest pain as having a physical/organic cause in both men and women.  The presence of stress and anxiety significantly altered students’ interpretation of women’s chest pain as being less likely to have a physical/organic cause but had little effect on the interpretation of men’s chest pain. | In the context of social stress, women’s chest pain is less likely to be perceived by medical students as having a physical/organic cause than men’s chest pain. | 11.5 |
| Schafer et al, 2016 | B | United Kingdom, single-site, videos and written vignettes | To evaluate medical students’ judgements of patients with chronic pain according to their ‘trustworthiness’, history of depression, and gender. | 29 medical students were shown videos of patients suffering from chronic pain and provided with vignettes that described patients’ levels of trustworthiness, and history of depression. Students were asked to estimate patients pain according to a numerical scale. | Medical students estimated men as being in more pain than women presenting with the same vignette. | Students assess men as being in more pain than women. | 11.5 |
| Tan et al, 2022 | B | United Kingdom, single-site, robotic mannikin | To evaluate differences in medical students’ clinical examination behaviours when palpating a simulated abdomen and receiving feedback on the extent of pain felt following palpation by observing computer generated facial expressions occurring on faces of different genders and ethnicities. | 16 medical students palpated a simulated abdomen and observed computer generated facial expressions that reflected the extent of pain that the palpation had caused. The timing and force of subsequent palpations were recorded and related to different face identities. | When medical students were the opposite gender to the computer-generated face, and the observed facial response to the palpation was deemed to be inappropriate, they applied the next palpation action more quickly than if the computer-generated face was the same gender as themselves.  For same gender interactions, male participants made relatively smaller increments and decrements in their peak forces to closely observe the corresponding pain facial expression changes when decisions were not apparent. | Medical student’s own gender influences the ability to interpret pain expressions in patients of the opposite gender, especially when there is a disconnect between their belief and the simulated pain response. | 11.5 |
| Rusconi et al, 2010 | B | Italy, single-site, written vignettes | To explore how variables such as physical signs of pain, facial expressions, age and gender affect medical students’ degree of belief in patients self-reported pain scores. | 160 medical students were given a series of vignettes of a patient presenting with a headache. Participants were provided with the patient’s name, age, gender, facial expression, presence/absence of physical pain signs and their self-reported pain score.  Students were asked to evaluate the patient’s pain, firstly categorising the extent to which each value on the pain rating scale was credible, partly credible or not credible, for the vignette that they had observed (discrete measure). Students were also asked to rank the values on the pain scale from most to least credible for the vignette that they had observed (graduated measure). Researchers then compared the students’ credibility ratings (discrete and graduated) with the patients’ self-reported pain scores. | Students were statistically more likely to report a lower pain rating (using the graduated measure) than patients’ self-reported pain if the patient was a woman. This difference was not observed using the discrete measure.  When physical signs of pain were present, students pain scores (using the graduated measure) compared more favourably to men’s self-reports of pain, than women’s. This difference was not observed using the discrete measure.  There was a trend not to trust the patient’s judgment if the patient is a woman when using the graduated measure. | When students used a graduated measure to estimate the credibility of patient’s pain, they judged women to be in less pain than they reported, even when physical signs of pain were present (i.e., there was a trend not to trust the patient’s judgment when the patient is a woman) | 9.5 |

NB: for clarity and to reflect the fact that studies referred to gender rather than biological sex, references to ‘male’ and ‘female’ patients have been altered to ‘woman/girl’ or ‘man/boy’.
